# A structural analysis of the splice-specific functional impact of the pathogenic familial hemiplegic migraine type 1 S218L mutation on Ca_v_2.1 P/Q-type channel gating

**DOI:** 10.1186/s13041-024-01152-z

**Published:** 2024-11-20

**Authors:** Anne-Sophie Sack, Gennerick J. Samera, Anna Hissen, Robert J. Wester, Esperanza Garcia, Paul J. Adams, Terrance P. Snutch

**Affiliations:** 1 Michael Smith Laboratories, University of British Columbia, 2185 East Mall, Vancouver, BC V6T 1Z4 Canada; 2 Djavad Mowafaghian Centre for Brain Health, University of British Columbia, 2215 Wesbrook Mall, Vancouver, BC V6T 1Z3 Canada; 3 Applied Genomics Centre, Kwantlen Polytechnic University, 12666 - 72 Ave, Surrey, BC V3W 2M8 Canada

**Keywords:** P/Q-type channel, FHM-1 mutation, Alternative splicing, Structural modeling, Gating

## Abstract

P/Q-type (Ca_v_2.1) calcium channels mediate Ca^2+^ influx essential for neuronal excitability and synaptic transmission. The CACNA1A gene, encoding the Ca_v_2.1 pore forming subunit, is highly expressed throughout the mammalian central nervous system. Alternative splicing of Ca_v_2.1 pre-mRNA generates diverse channel isoforms with distinct biophysical properties and drug affinities, which are differentially expressed in nerve tissues. Splicing variants can also affect channel function under pathological conditions although their phenotypic implication concerning inherited neurological disorders linked to CACNA1A mutations remains unknown. Here, we quantified the expression of Ca_v_2.1 exon 24 (e24) spliced transcripts in human nervous system samples, finding different levels of expression within discrete regions. The corresponding Ca_v_2.1 variants, differing by the presence (+) or absence (Δ) of Ser-Ser-Thr-Arg residues (SSTR) in the domain III S3-S4 linker, were functionally characterized using patch clamp recordings. Further, the + /ΔSSTR isoforms were used to demonstrate the differential impact of the Familial Hemiplegic Migraine Type 1 (FHM-1) S218L mutation, located in the domain I S4-S5 linker, on the molecular structure and electrophysiological properties of Ca_v_2.1 isoforms. S218L has a prominent effect on the voltage-dependence of activation of +SSTR channels when compared to ΔSSTR, indicating a differential effect of the mutation depending on splice-variant context. Structural modeling based upon Cav2.1 cryo-EM data provided further insight reflecting independent contributions of amino acids in distant regions of the channel on gating properties. Our modelling indicates that by increasing hydrophobicity the Leu218 mutation contributes to stabilizing a structural conformation in which the domain I S4-S5 linker is oriented alongside the inner plasma membrane, similar to that occurring when S4 is translocated upon activation.The SSTR insertion appears to exert an influence in the local electric field of domain III due to an change in the distribution of positively charged regions surrounding the voltage sensing domain, which we hypothesize impacts its movement during the transition to the open state. In summary, we reveal molecular changes correlated with distinct functional effects provoked by S218L FHM-1 mutation in hCa_v_2.1 splice isoforms whose differential expression could impact the manifestation of the neurological disorder.

**Supplementary Information:**

The online version contains supplementary material available at 10.1186/s13041-024-01152-z.

## Introduction

Voltage-gated Ca_v_2.1 (P/Q-type) channel function is essential to excitation-secretion coupling and regulating neurotransmitter and hormone release [1, 2]. In mature excitatory central synapses, Ca^2+^ influx through Ca_v_2.1 mediates synaptic vesicle exocytosis and contributes to evoked glutamate release [1]. P/Q-type channels are heteromeric protein complexes formed by the Ca_v_2.1 α_1_ subunit, which determines biophysical properties and pharmacological sensitivity, and associated β and α_2_δ ancillary subunits that regulate membrane targeting and other functional properties [3].

The CACNA1A gene encoding the pore forming α_1_ subunit of the Ca_v_2.1 contains at least 47 exons [4]. Alternative splicing sites located in key functional domains of rodent and human Ca_v_2.1 channels [5] confer distinct biophysical properties as well as defining the pharmacological distinction between native P- and Q-types [6, 7]. As P/Q-type currents are tightly coupled to neurotransmitter release at fast CNS synapses [8, 9], splice variation may be critical to influencing temporal precision and plasticity of synaptic responses and hence information flow in neuronal circuits [10, 11].

Missense mutations in CACNA1A are associated with a wide range of neurological and neurodevelopmental disorders including episodic ataxia, epileptic encephalopathy and congenital migraine [12–14]. Here, we focus on CACNA1A mutations underlying Familial Hemiplegic Migraine type-1 (FHM-1), a rare autosomal condition with phenotypes ranging from migraine with aura to hemiparesis and progressive cerebellar ataxia [15, 16]. Amongst the dozens of described FHM-1 missense mutations, S218L causes a severe clinical phenotype that includes epileptic seizures and ataxia, and can result in premature death [17–19]. The functional impact of the S218L FHM-1 mutation has been thoroughly studied in recombinant human Ca_v_2.1 channels [20, 21] as well as in transgenic knock-in mice [22–25]. To date however, the structural implications of the S218L substitution in the Ca_v_2.1 domain I S4-S5 linker have not been reported.

Alternative splicing can modify the severity of alterations provoked by disease-associated mutations in CACNA1A gene. For example, a frame shift resulting in a stop codon or the insertion of a pentanucleotide at the beginning of exon 47 generates short (Δ47) or long (+47) C-terminal splice variants [5, 7] respectively, which show distinctive features of the S218L mutation concerning voltage-dependent gating, recovery from inactivation and Ca^2+^-dependent regulation [20]. A variant-dependent effect in these two carboxyl tail isoforms has also been reported for the pathogenic mutations Y1384C, located in transmembrane segment domain III S5 [26] as well as mutations R1667W and S1799L, located in domain IV S4 and domain IV S6, respectively [27].

Initial homology-based Ca_v_ modeling studies have provided clues concerning structural alterations introduced by amino acid substitutions in the Ca_v_2.1 channel as a result of pathogenic missense mutations [26–30]. Among the molecular changes provoked by point mutations of clinical significance, alterations include the loss of electrostatic interactions with the emergence of hydrophobic interactions not present in the wild-type channel [27], steric distortion of helical structure [29, 30], loss of hydrogen bonds between adjacent segments [27, 29], and changes in electrostatic potential [26].

**Fig. 1:**
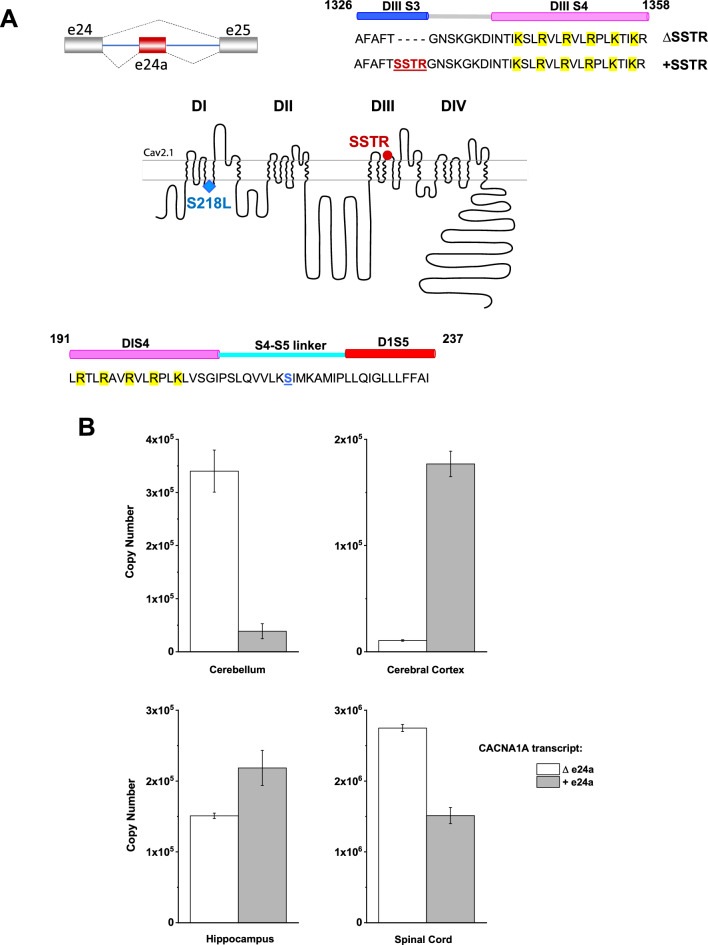
Expression of two CACNA1A isoforms generated by alternative splicing of exon 24a in human nervous tissue. Upper panel **A** shows a schematic representation of the cassette exon 24a (left) and the amino acid sequences of the isoforms ΔSSTR and + SSTR (right), resulting from the alternative splicing event. The sequence shown corresponds to residues 1326 to 1358; the inclusion of the tetrapeptide (underlined red) occurs at the limit of the terminal portion of S3 and the extracellular loop connecting with S4 in domain III. Highlighted residues in yellow correspond to the S4 gating charges. The topology diagram of the hCa_v_2.1 α_1A_ subunit (middle) shows the location of the FHM-1 S218L mutation (blue diamond in the S4-S5 intracellular loop of domain I) and the tetrapeptide SSTR encoded by exon 24a (red circle domain III). Lower panel A: amino acid sequence of the S4-S5 linker of domain I (residues 191–237), with residue Ser218 labeled in blue, is shown below the topology diagram. Amino acid numbers correspond to the sequence of the clone used for generating the Cav2.1 cryo-EM structure (PDB: 8X90) used in our study for molecular modeling. **B** shows the comparison of copy number between transcript isoforms Δe24a and + e24a, obtained using qRT-PCR. Bar graphs represent the copy number values from different human tissue samples (see Table 1), derived from standard curves using qPCR CTs in triplicate. Error bars indicate the variability among replicates. The Δe24a variant is more abundant in the cerebellum and spinal cord, whereas + e24a is the predominant variant in the cerebral cortex

However, it remains to be described whether the underlying molecular mechanism of splice-specific functional alterations involves a concerted action of conformational changes due to the combination of mutations and alternative splicing. In this study we address this issue using as a paradigm the S218L mutation expressed in splice isoforms whose functional characteristics have not been reported. Initially, we examined *Cacna1a* mRNA transcripts from adult rat nervous tissue and identified an alternative splice variant with high level of expression in the rat spinal cord compared to the forebrain. The transcript contains exon 24a and corresponds to a Ca_v_2.1 variant [31] with an insertion of the tetrapeptide SSTR in the S3-S4 linker of domain III (Fig. 1A) albeit with no previous functional data reported. Given the disease implications of splice-variant differential effects of FHM-1 mutations, we further quantified the expression levels of the e24 alternative transcripts from different regions of human nervous system. We also examined the electrophysiological properties of the hCa_v_2.1 +SSTR recombinant variant and the context-dependent effect of the S218L substitution expressed in + and ΔSSTR isoforms. Finally, employing structural modeling using the recently described cryo-EM structure of Ca_v_2.1 [32] and patch-clamp recordings, we sought to find a structure-guided mechanistic interpretation of the splice-dependent impact of pathogenic mutations on hCa_v_2.1 functional properties.

## Methods

### Quantitative real-time PCR

Human cDNA samples from 4 regions of the central nervous system (cerebellum, cerebral cortex, hippocampus and spinal cord) were sourced from BioChain (Newark, CA, USA), with each region obtained from different individuals of different ages, sexes, and geographical background with no diagnosed neurological conditions (Table 1).

**Table 1:** Demographic information of central nervous tissue cDNA Donors

Tissue source (Cat. #)	Donor age (yrs)	Donor sex	Donor background
Cerebellum (C1234039)	82	Female	Caucasian
Cerebral Cortex (C1234042-10)	72	Female	Caucasian
Hippocampus (C1234052-10)	66	Male	Asian
Spinal cord (C1234234)	44	Male	Asian

Two hydrolysis probes were used for quantification of CACNA1A expression (Table 2): a probe that targets the inclusion of e24a (custom; CAV2.1-P-SSTR), and a probe that targets the exclusion (custom; CAV2.1-M-SSTR). Hydrolysis probes targeting reference genes *CYC1* (ThermoFisher; Hs00357717_m1) and *TBP* (ThermoFisher; Hs00427620_m1) were used to validate sample quality. Custom probes were designed using Primer Express Software v3.0.1 (Applied Biosystems; 4,363,991) and custom Python scripts. Gene expression assays were designed with unlabeled PCR primers and a hydrolysis probe with FAM and minor groove binder (MGB) on the 5’ end and BkFQ quencher on the 3’ end.

**Table 2:** Custom assay sequences

Name	Forward primer sequence (5′-3′)	Reverse primer sequence (5′-3′)	Probe sequence (5′-3′)
CAV2.1-P-SSTR	GGCCCTGGTAGCCTTTGC	TGATGTCTTTTCCTTTGCTATTGC	TTCACGAGCAGTACACG
CAV2.1-M-SSTR	CCTACTTCCGTGACCTCTGGAA	CGGAGGACTCGGAGGGATTTAA	TGCCTTCACTGGCAAT

Absolute quantification of + /Δe24a transcripts was performed by creating a standard curve with a dilution series of plasmids containing each respective transcript of interest. The standard curve for CAV2.1-P-SSTR and CAV2.1-M-SSTR was generated with a series of four 1:5 dilutions in triplicate starting from 22.14 ng/μL and 14.30 ng/μL, respectively (Table 3). The correlation coefficient of standard curves exceeded 0.99 for both CAV2.1-P-SSTR (0.997) and CAV2.1-M-SSTR (0.997).

**Table 3:** Summary qPCR standard curve parameters

Assay	Slope	R^2^	Y-intercept	% Efficiency
CAV2.1-P-SSTR	−3.9	0.997	20.016	91.024
CAV2.1-M-SSTR	−3.558	0.997	20.841	80.461

Quantitative PCR (qPCR) reactions were set up in triplicate using TaqMan™ Fast Advanced Master Mix (Applied Biosystems; 4,444,556) in MicroAmp Fast Optical 96-Well Reaction Plates (Applied Biosystems; 4,346,907) and performed on a QuantStudio 7 Real-Time PCR System (Applied Biosystems; 4,485,701). Reactions were prepared in 10 μL volumes with each reaction containing 1 ng of cDNA. The qPCR cycling conditions were as follows: initial denaturation at 95 °C for 20 s, followed by 40 cycles of denaturation at 95 °C for 1 s, and annealing and extension at 60 °C for 20 s.

### Recombinant Ca_v_2.1 plasmid preparation for heterologous expression

The biophysical properties of four recombinant isoforms of the human Ca_v_2.1 channel were studied: human Ca_v_2.1 (Transcript variant 3; NCBI Ref. Sequence NM_001127221.2, hereinafter referred to as wt ΔSSTR), FHM-1 mutant S218L in the ΔSSTR background [20], and two alternative splice isoforms; wt +SSTR, and its corresponding mutant S218L +SSTR. Briefly, the isoform wt +SSTR was created as follows: a 2,751 bp *Eco*RI fragment of wt ΔSSTR was subcloned into pBlueScript KS (+) and the SSTR protein coding DNA insertion introduced using the NEB Q5 Site-Directed Mutagenesis kit (E0554S) and the primers hA-SSTR-Q5SDM-F (tacacgTGGCAATAGCAAAGGAAAAG) and hA-SSTR-Q5SDM-R (ctgctcGTGAAGGCAAAGGCTACC) using the manufacturers recommended conditions. Following mutagenesis, the correct 2,763 bp *EcoR*I region, containing the 12 bp insertion (GAGCAGTACACG) [31], was identified in candidate clones using a *Pml*I digestion followed by sequencing confirmation (pBS-hA-SSTR). A 2,763 bp *Eco*RI fragment was excised from pBS-hA-SSTR, purified, and then ligated to a purified 9,747 bp *Eco*RI fragment of wt ΔSSTR. Candidate clones were screened for correct orientation using restriction digests and sequencing. Once correct orientation was confirmed, full sequencing of the insert was performed to verify addition of the SSTR coding insertion. Human wild-type Ca_v_2.1 containing the SSTR hereinafter referred to as wt ΔSSTR.

The cDNA S218L +SSTR CACNA1A variant was prepared by ligation using the FHM-1 mutant S218L in the ΔSSTR background [20] clone and the wt +SSTR clone. Briefly, both plasmids were subjected to a double digestion with XhoI (NEB catalog no. R0146S) and BsaBI (NEB catalog no. R0537S). Fragments were separated using Low Melting Point Agarose (ThermoFisher catalog no. 16520050) excised, purified and ligated.

Ligation product was transformed into *E. coli*, and incubated overnight at 37 °C. Colonies were inoculated into 4 mL of 100 µg/mL carbenicillin LB Medium (MP Bio catalog no. 3002–022) and incubated overnight at 37 °C shaking at 250 rpm. Plasmid DNA was purified using the QIAprep Spin Miniprep Kit (Qiagen catalog no. 27104) and subject to sequencing to verify the presence of both the S218L mutation and +SSTR isoform. Additionally, restriction sites were sequenced to confirm proper ligation.

### Cell culture and transfection

HEK293-F cells (Invitrogen 11,625–019) were maintained at 37 °C in a humidified incubator with an atmosphere of 95% air-5% CO_2_. Growth medium (Dulbecco’s modified Eagle’s medium; Gibco 12,800–017) was supplemented with 10% heat-inactivated fetal bovine serum (FBS; Gibco12483020) and 1% non-essential amino acids (GIBCO 11140–050). Cells were seeded on poly-D-Lysine coated (0.1 mg/ml; Sigma-Aldrich P7886) glass coverslips ~ 26 h before transfection. Cotransfection of each hCa_v_2.1 α_1_ subunit with ancillary subunits β_4_ and α_2_δ_1_, and green fluorescent protein (GFP) at a molar ratio 1:1:1:0.1 was performed using Turbofect Reagent (Thermo Scientific 01270256), following the procedure from the manufacturer.

### Electrophysiology

Approx. 18 to 21 h after transfection coverslips were transferred to a recording chamber and continuously perfused with external solution at a flow rate of 1.4–1.5 ml/min using a valve-controlled gravity-driven perfusion system (ALA VM8, Scientific Instruments). Recording pipettes were made from 1.5 mm OD borosilicate glass capillaries (Sutter Instruments Co) using a P-97 Flaming/Brown horizontal puller (Sutter Instruments Co.) and fire-polished with a MF-900 microforge (Narishige Group). Electrode resistances ranged from 2.7 to 3.8 MΩ when filled with an internal solution containing (in mM): 105 CsMeSO_4_, 25 TEA-Cl, 11 EGTA, 10 HEPES, 1 CaCl_2_, 5 ATP-Mg and 0.4 GTP-Na, 3 Tris-phosphocreatine and 3 Na_2_-phosphocreatine (pH 7.2). The extracellular bathing solution contained (in mM): 2 CaCl_2_, 1 MgCl_2_, 92 CsCl, 40 TEA-Cl, 10 HEPES and 10 Glucose (pH 7.4). Osmolality was adjusted with D-Mannitol to 290mOsmol/Kg and 305 mOsmol/Kg, for the internal and external recording solution, respectively.

Whole-cell patch-clamp recordings were at room temperature (22–24 °C) using an Axopatch 200B (Molecular Devices) amplifier and acquired with a Digidata 1322A System (Molecular Devices). Macroscopic Ca^2+^ currents were typically filtered at 2 kHz and digitized at 10 kHz using pClamp 9 software, except for the deactivation protocol (filtered at 5 kHz and sampled at 100 kHz and 50 kHz, respectively). Series resistance was compensated at 75–80%. Clampfit Analysis Module in the pCLAMP 11 Software Suite was used to measure and fitting current traces; data plotting and statistical analysis were carried out using and OriginLab 9.7.0.188 and Graph Pad-Prism 10.0.2.

The current–voltage (I-V) relationship was obtained with a series of 90 ms step depolarizations between −60 to + 35 mV with increments of 5 mV, from a holding potential of −90 mV. Maximum peak current amplitudes I_Ca_ were divided by the cell membrane capacitance to obtain current density (pA/pF), and plotted against the test pulse potential (Vm). Data points were fitted with a modified Boltzmann equation:

$${\text{I}}_{{{\text{Ca}}}} = {\text{ G }}\left( {{\text{Vm }} - {\text{ Er}}} \right) \left( {{1 } + {\text{ exp }}\left[ {\left( {{\text{Vm }} - {\text{ V}}_{{{5}0}} } \right) \, /{\text{ k}}} \right]} \right)$$

where I_Ca_ is the peak value at test potential Vm, Er is the apparent reversal potential, V_50_ is the voltage for half-maximal activation, k is the slope factor, and G is the conductance.

Inactivation properties were examined with 5 s conditioning pre-pulses at voltages ranging from −120 to + 10 mV (in 10 mV increments), followed by an 80 ms test pulse at 0 mV. Currents recorded during the test pulses were normalized to the maximal value, plotted as a function of the pre-pulse membrane potential, and fitted with a Boltzmann equation to obtain the steady-state inactivation curves:

$${\text{I }}/{\text{ I}}_{{{\text{max}}}} = \, ({1 }/ \, \left( {{1 } + {\text{ exp }}\left[ {\left( {{\text{Vm }}{-}{\text{ V}}_{{{5}0}} } \right) \, /{\text{ k}}} \right]} \right)$$

where I is the peak current obtained after various inactivating prepulses, I_max_ is the peak current after the prepulse at −120 mV, V_50_ is the voltage for half-maximal activation and k is the slope factor.

To obtain the activation curves, conductance values as a function of membrane potential were fitted using the Boltzmann equation:

$${\text{G}}/{\text{G}}_{{{\text{max}}}} = \, ({1 }/ \, \left( {{1 } + {\text{ exp }}\left[ {\left( {{\text{Vm }}{-}{\text{ V}}_{{{5}0}} } \right) \, /{\text{ k}}} \right]} \right)$$

Data is shown as mean ± SEM. Comparison of the mean values of voltage dependence parameters and current density were made between all four conditions using a one-way ANOVA with multiple comparison analyses using Tukey test. The Brown-Forsythe test was used to test for equal variance assumption, and Welch’s ANOVA was used when appropriate with Dunnett’s T3 multiple comparison. All statistical analysis was performed using Graph Pad-Prism 10.0.2. Statistical difference was tested at a 0.05 level of significance.

### Molecular modeling

Initially, we employed a homology-based approach using the Ca_v_2.2 cryo-EM structure [33] as a template for our study. However, the recent publication of the Ca_v_2.1 cryo-EM structure [32] allowed us to directly examine the S218L mutation and SSTR tetrapeptide splice variation. Notably, the effects of the S218L mutation and SSTR insertion were well predicted by the homology model and the structures were highly similar (data not shown). The apo structure of Ca_v_2.1 (PDB: 8X90) was uploaded into MOE Molecular Operating Environment with Amber10: EHT force field parameters and prepared for analysis using the structure preparation application that assigns ionization states, positions hydrogens, caps N or C-termini and neutralizes residues in structural gaps (2022.02 Chemical Computing Group UCL, 910–1010 Sherbrooke St. W., Montreal, QC, H3A 2R7, Canada, 2023). The structural model of the S218L mutation in the domain I S4-S5 intracellular linker was generated using the residue scan function. The SSTR insertion in domain III between amino acids T1330 and G1331 was modeled using both de novo and PDB structures as a template. Top loops were ranked based on their initial coarse scores to assess the quality of the loop using the backbone atoms and a final score based on potential energy using the Generalized Born/Volume Integral (GB/VI) solvation of the protein [34]. Loops were generated with different combinations of anchoring residues surrounding the SSTR insertion and were compared based on the final energy scores and protein geometry scores. The degree of sequence similarity with existing PBD structures was also considered when scoring top loops. The top five loops were compared with both the initial cryo-EM structure as well as modeled ΔSSTR loops as a control to account for differences arising from molecular modeling that are not related to the insertion itself. Protein patches were created using MOE to analyze hydrophobicity and charged regions.

In order to visualize the location of the S218L mutation in the context of the plasma membrane, a computed simulation model of a lipid bilayer was generated. CHARMM**-**GUI HMMM Builder [35, 36] was used to insert the protein within a general neuronal plasma membrane, based on Ingólfsson et al. [37]. The composition of the plasma membrane used for visualization of the protein-membrane model is listed in Table 4.

**Table 4:** Full composition of membrane lipids* used for CHARMM-GUI

Membrane LIPID		Outer leaflet count	Inner leaflet count
Phosphatidylcholine (PC)			
	DOPC	221	118
	DPPC	531	284
	OIPC	59	32
	OUPC	42	22
	PAPC	463	247
	PFPC	59	32
	POPC	868	463
	PUPC	169	90
Phosphatidylethanolamine (PE)			
	OAPE	68	127
	OIPE	14	27
	OUPE	72	133
	PAPE	312	574
	POPE	127	234
	PUPE	500	922
Sphingomyelin (SM)			
	BNSM	108	27
	DPSM	581	143
	PBSM	132	32
	POSM	71	17
Phosphatidylserine (PS)			
	DPPS		46
	OUPS		65
	PAPS		261
	PUPS		326
Phosphatidylisonitols (CER)			
	PAPI		121
	PIPI		48
	POPI		121
	PUPI		194
Diacylgylcerol (DAG)			0.5
	PADG	25	25
	PODG	13	13
Cholesterol (CHOL)			
	CHOL	4431	4222

^*^According to IIngólfsson et al. [37]

## Results

### CACNA1A ∆e24a and + e24a transcripts are differentially expressed in the human nervous system.

Absolute quantification of splice variant transcripts was performed employing a standard curve and related to copy number for comparison of +/Δe24a in human brain samples (Fig. 1B; Table 5). The expression of reference genes, *TBP* and *CYC1*, was stable across samples (Table 6). In individual brain regions, the expression of +e24a and Δe24a transcripts varied. The Δe24a was found more abundantly expressed in cerebellum and spinal cord while cerebral cortex and hippocampus expressed significantly higher levels of + e24a transcripts. These results suggest the expression of +SSTR and ΔSSTR Ca_v_2.1 channels varies across different brain regions, consistent with previous studies showing the differential expression of Ca_v_2.1 splice variants in human brain [5, 38].

**Table 5:** Copy number for ∆e24a and + e24a in human brain samples

Variant	Cerebellum	Cerebral cortex	Hippocampus	Spinal Cord
∆e24a (copies) (% relative proportion)	340,112 ± 39,555 (90%)	10,660 ± 676 (6%)	150,900 ± 3834 (41%)	2,747,953 ± 50,298 (65%)
+ e24a (copies) (% relative proportion)	38,767 ± 14,008 (10%)	176,854 ± 11,990 (94%)	218,503 ± 24,753 (59%)	1,511,899 ± 113,408 (35%)

**Table 6:** Mean PCR cycle threshold (Ct) values for reference genes *CYC1* and *TBP* across brain regions

Reference gene	Cerebellum	Cerebral cortex	Hippocampus	Spinal cord
*TBP*	28.5 (28.41–28.49)	32.0 (31.78–32.13)	29.0 (28.84–29.03)	25.8 (25.71–25.84)
*CYC1*	24.6 (24.60–24.66)	26.3 (26.26–26.38)	23.9 (23.83–23.90)	21.7 (21.80–21.74)

### Distinct splice-variant dependent effects of the FHM-1 S218L mutation on Ca_v_2.1 biophysical properties

**Fig. 2:**
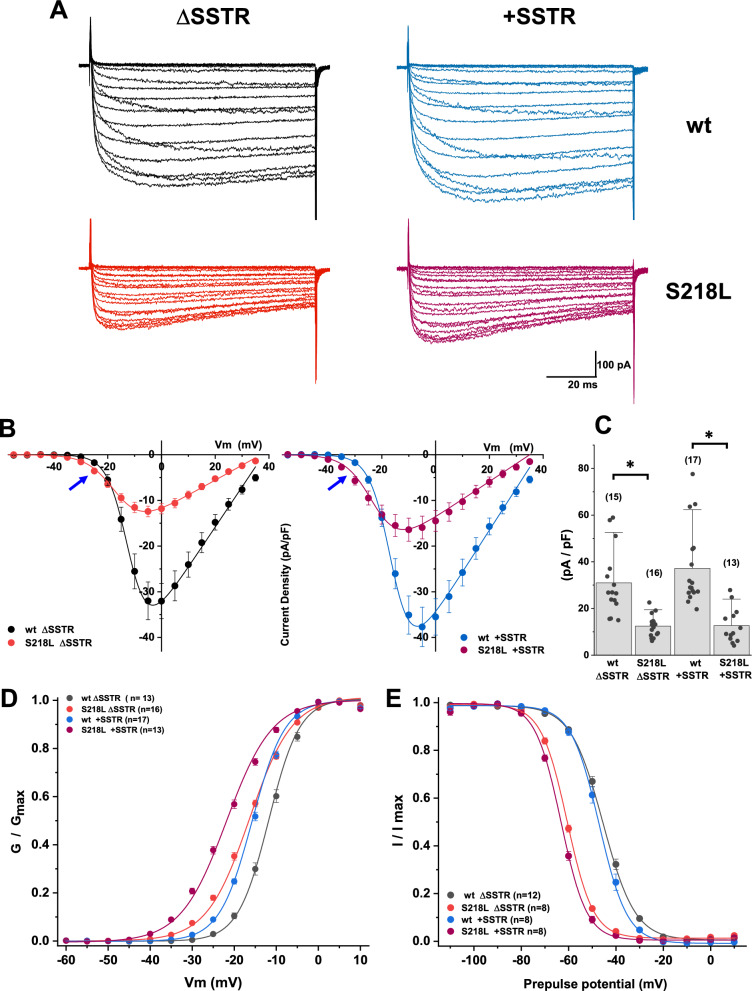
Inclusion of exon 24a enhances the functional impact of S218L mutation on Cav2.1 activation gating. **A**. Macroscopic Ca^2+^ currents evoked by 90 ms voltage steps between -60 and + 35 from a holding potential of -90 mV, recorded from HEK293F cells expressing wt hCa_v_2.1 channel splice isoform ΔSSTR (top left), wt +SSTR (top right), or S218L mutant channels in the corresponding splice variant background (bottom panels). Current density *vs* voltage relationships (**B**) show that alternatively spliced wild type +SSTR channel displays a negative shift in activation threshold, relative to the wild type ΔSSTR. I-V curves show the distinctive effects of the S218L mutation, such as reduced current density and a hyperpolarizing shift of the activation voltage. Mutation-induced effects were similar between the two splice isoforms; however, S218L +SSTR mutant channel (Panel B, right) displayed a shallower I-V curve slope factor and a further shift towards more negative potentials (see blue arrows), compared to the S218L ΔSSTR (panel B, Left). Bar graph (**C**) shows a comparison of the averaged current density (pA/pF) values at the peak of the I-V curve. Both S218L mutant channels display reduced current density, regardless of channel splice isoform. The number of cells recorded is given in parentheses; the asterisk indicates statistical significance < 0.0001 of mutant channel values, relative to their respective wt. Voltage dependence of activation (**D**) and inactivation (**E**) plots revealed that activation half-point (V_50_) and slope factor (k) are significantly different between the two wild type splice isoforms (blue dots **D**), whereas steady-state inactivation curves were nearly identical (black dots vs blue dots, **E**) (Table 7). The activation curve is shallower for the mutant channels with a prominent hyperpolarizing shift of the activation voltage to even more negative potentials (**D**). S218L mutant channels display a similar difference (~ 15 mV) in half-maximal inactivation (**E**), relative to their corresponding splice isoform

**Fig. 3:**
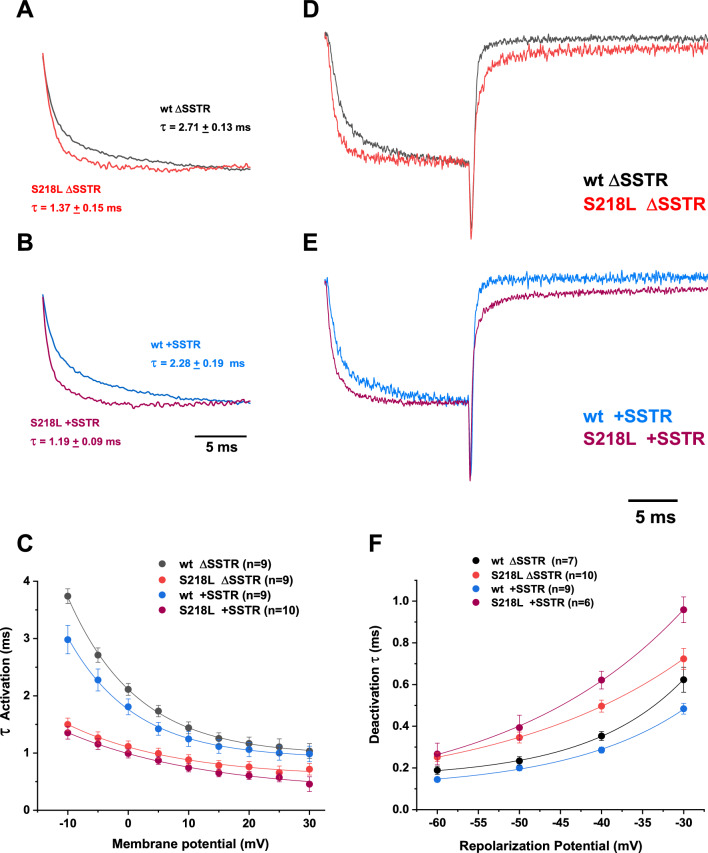
S218L FHM-1 mutation alters the kinetic properties of P/Q-type currents mediated by + /Δ SSTR isoforms. Superimposed normalized recordings of peak currents from SSTR (**A**) and +SSTR (**B**) isoforms show the difference in the activation time course between wild type and S218L channels, with the onset of activation distinctively faster in the mutant channels (S218L ΔSSTR, red trace in A; S218L +SSTR purple trace in B). The average activation time constant values at the peak of the I-V curve is shown next to the corresponding traces. Mean activation time constants were plotted against membrane potential (**C**), showing that mutant channels display a shallower voltage dependence than the wild types. To examine channel deactivation, currents were evoked by a brief depolarizing test pulse from a holding of −100 to −5 mV, and membrane potential was repolarized to various membrane potentials to record tail currents. Exemplary recordings corresponding to repolarization to −40 mV from each pair of wt (black trace, **D**; blue trace, **E**) vs mutant isoforms (red trace, **D**; purple trace, **E**) were normalized and overlapped, showing that both mutant channels not only activate faster, but also take longer to close when the membrane is repolarized, relative to wild type. The decay phase of the tail currents was fitted with a single exponential to obtain deactivation time constants, plotted as a function of the repolarization potential (**F**). Deactivation voltage dependence was more pronounced in the wild type isoforms (see text for details)

In order to compare the functional effects of S218L mutation on alternatively spliced variants, we examined macroscopic currents in HEK293 cells expressing ΔSSTR or +SSTR wild type isoforms (upper panels Fig. 2A) as well as their corresponding mutant isoforms (lower panels Fig. 2A). Examples of representative currents recorded over a range of test potentials using Ca^2+^ as the charge carrier are shown in Fig. 2A, and current–voltage profile was analyzed by plotting the average current density as a function of membrane potential (Fig. 2B). As previously described, S218L mutation leads to a reduction in maximal current density (Fig. 2C): wt ΔSSTR 30.96 ± 3.73 pA/pF, n = 15; S218L ΔSSTR 12.46 ± 1.16 pA/pF, n = 16; wt +SSTR 37.20 ± 4.07 pA/pF, n = 17; S218L +SSTR 12.68 ± 2.09 pA/pF, n = 13. One-way ANOVA F_(3,57)_ = 16.86, p < 0.0001, post hoc Tukey test wt ΔSSTR vs. S218L ΔSSTR p = 0.0005 and wt +SSTR vs. S218L +SSTR p < 0.0001. Close examination of the I-V curves revealed higher current density at membrane potentials negative to -20 mV (blue arrows, Fig. 3B), particularly for the S218L +SSTR mutant channel.

Voltage-dependence gating was analyzed using the normalized conductance plots (Fig. 2D) and the steady state inactivation curves (Fig. 2E). In wildtype channels, the main consequence of +SSTR inclusion was to shift the voltage dependence of channel activation to more negative potentials. Parameters obtained from the best fit of a single Boltzmann equation to the experimental data (see Table 7) revealed that half-maximal activation (V_50_) values were significantly different between the two wild type splice isoforms (wt ΔSSTR V_50_: −11.80 ± 0.46, n = 13; wt +SSTR V_50_: −15.44 ± 0.30, n = 17, one-way ANOVA F_(3,54)_ = 114.7 p < 0.0001, post hoc Tukey test wt ΔSSTR vs. wt +SSTR p < 0.0001), no significant difference in the slope factor (*k*) (wt ΔSSTR *k*: 3.65 ± 0.08, n = 13; wt +SSTR *k*: 3.99 ± 0.06, n = 17), and no significant changes were observed in the steady-state inactivation (wt ΔSSTR V_50_: −45.06 ± 0.73, n = 12; wt +SSTR V_50_: −47.16 ± 0.64, n = 8; wt ΔSSTR *k*: 6.42 ± 0.12, n = 12; wt +SSTR *k*: 6.08 ± 0.18, n = 8).

**Table 7:** Activation and steady-state inactivation parameters

hCa_v_2.1	Activation V_50_ (mV)	Activation *k*	N	Inactivation V_50_ (mV)	Inactivation *k*	N
wt ΔSSTR	−11.80 ± 0.46	3.65 ± 0.08	13	−45.06 ± 0.73	6.42 ± 0.12	12
S218L ΔSSTR	−16.78 ± 031***	5.21 ± 0.16***	16	−60.47 ± 0.37***	5.28 ± 0.18**	8
wt +SSTR	−15.44 ± 0.30^**###**^	3.99 ± 0.06	17	−47.16 ± 0.64	6.08 ± 0.18	8
S218L +SSTR	−22.05 ± 0.44***	5.70 ± 0.04***	12	−62.90 ± 0.43***	5.24 ± 0.31*	8

Summary table showing mean values ± SEM of activation and inactivation voltage dependence parameters. N = sample size. P values listed below correspond to results from one-way ANOVA with post hoc Tukey test

^***^: P value < .0001 indicate statistical significance of S218L mutant voltage dependence values as compared to the corresponding wild type

^**###**^: *P* value < .0001 from the statistical comparison between activation V_50_ of wild type channels

^**^: P = 0.0009 from statistical comparison between S218L ΔSSTR and wt ΔSSTR

The significance level obtained by comparing activation V_50_ and *k* values between S218L mutant channels was *P* < .0001, and *P* = 0.0108, respectively. The significance level obtained for *k* values between wild type channels was P = 0.0980

^*^: *P* value = 0.0364 from statistical comparison between S218L +SSTR and corresponding wildtype +SSTR

Comparison between the two wild-type isoforms (V_50_ *P* = 0.0821; *k P* = *0.5809*) indicates no statistical difference of inactivation parameters. Comparison between the two mutant channels shows no significant difference in V_50_ (*P* = 0.0616), or *k (P* = *0.9*993

The S218L mutant shifted the activation curves towards more negative values compared to their wild type splice isoform counterparts (Fig. 2D), with significantly different midpoint values (wt ΔSSTR V_50_: -11.80 ± 0.46, n = 13; S218L ΔSSTR V_50_: −16.78 ± 0.31, n = 16; wt +SSTR V_50_: −15.44 ± 0.30, n = 17; S218L +SSTR V_50_: −22.05 ± 0.44, n = 12; one-way ANOVA F_(3,54)_ = 114.7 p < 0.0001, post hoc Tukey test wt ΔSSTR vs. S218L ΔSSTR p < 0.0001, wt +SSTR vs. S218L +SSTR p < 0.0001). The conductance-voltage relation was steeper in both wild type splice isoforms compared to the mutants, as indicated by the slope factor (wt ΔSSTR *k*: 3.65 ± 0.08, n = 13; S218L ΔSSTR *k*: 5.21 ± 0.16, n = 16; wt +SSTR *k*: 3.99 ± 0.06, n = 17; S218L +SSTR *k*: 5.70 ± 0.04, n = 12, one-way ANOVA F_(3,54)_ = 83.52 p < 0.0001, post hoc Tukey test wt ΔSSTR vs. S218L ΔSSTR p < 0.0001, wt +SSTR vs. S218L +SSTR p < 0.0001).

The effect of S218L mutation on the steady-state inactivation of the +SSTR variant paralleled the effect elicited on ΔSSTR (Fig. 2E). The most striking effect was a hyperpolarizing shift (wt ΔSSTR V_50_: −45.06 ± 0.73, n = 12; S218L ΔSSTR V_50_: −60.47 ± 0.37, n = 8; wt +SSTR V_50_: −47.16 ± 0.64, n = 8; S218L +SSTR V_50_: −62.90 ± 0.43, n = 8, one-way ANOVA F_(3,32)_ = 217.7, post hoc Tukey test wt ΔSSTR vs. S218L ΔSSTR p < 0.0001, wt +SSTR vs. S218L +SSTR p < 0.0001). The effect of the mutation on the inactivation slope factor was more pronounced for the ΔSSTR variant (wt ΔSSTR *k*: 6.42 ± 0.12, n = 12; S218L ΔSSTR *k*: 5.28 ± 0.18, n = 8), although the change on the +SSTR was also significant (wt +SSTR *k*: 6.08 ± 0.18, n = 8; S218L +SSTR *k*: 5.24 ± 0.31, n = 8, one-way ANOVA F_(3,32)_ = 9.572 p = 0.0001, post hoc Tukey test wt ΔSSTR vs. S218L ΔSSTR p = 0.0009 and wt +SSTR vs. S218L +SSTR p = 0.0364). We observed that, as a consequence of the hyperpolarizing shift in the activation of S218L mutant channels, the membrane potential at which the maximum window current is observed was ~ 13 mV more negative than that of wild type channels in both splice backgrounds (supplementary Fig. 1). Our data is consistent with a study using brainstem slices from transgenic mice harboring the S218L mutation [22], where presynaptic calcium currents recorded from the calyx of Held displayed a hyperpolarizing shift in the window current.

A comparison of the time-dependent current increase at the peak of the IV curve for each isoform is shown in Fig. 3 (A, B). The time course of Ca^2+^ current activation was similar between the two splice isoforms, and S218L mutation results in a faster current rise, regardless of splice variant (wt ΔSSTR = 2.71 ± 0.12 ms, n = 9; S218L ΔSSTR = 1.37 ± 0.15 ms, n = 9; wt +SSTR = 2.28 ± 0.19 ms, n = 9; S218L +SSTR = 1.19 + 0.09 ms, n = 10, one-way ANOVA F_(3,33)_ = 27.08 p < 0.0001, post hoc Tukey test wt ΔSSTR vs. S218L ΔSSTR p < 0.0001 and wt +SSTR vs. S218L +SSTR p < 0.0001).

Activation time constants, obtained from exponential fits to the rising phase of macroscopic currents, decrease sharply with an increase in depolarizing steps (Fig. 3C). The voltage dependence of the activation kinetics were less pronounced in the mutant channels than their respective wild type splice isoform: wt ΔSSTR 11.66 ± 0.86 mV per e-fold change n = 9, S218L ΔSSTR 16.56 ± 1.22 mV per e-fold change n = 9; wt +SSTR 12.05 ± 0.85 mV per e-fold change n = 9, S218L +SSTR 20.33 ± 1.53 mV per e-fold change n = 10, one-way ANOVA F_(3,33)_ = 12.58 p < 0.0001, post hoc Tukey test wt ΔSSTR vs. S218L ΔSSTR p = 0.0311 and wt +SSTR vs. S218L +SSTR p < 0.0001).

An impact of the S218L mutation on current kinetics was also observed concerning the rate of deactivation. To examine the kinetics of channel closing, a 15 ms depolarization to −5 mV was followed by repolarization to a range of membrane potentials between −60 to −20 mV; the decay of repolarization-induced tail currents was analyzed by fitting a single exponential to obtain time constants. Representative traces showing the slowed deactivation of mutant channels, compared to their respective wild type splice isoforms, are shown on Fig. 3D, E; decay time constants of the tail currents evoked by repolarizing to −40 mV were significantly different (wt ΔSSTR = 0.35 ± 0.02, n = 7; S218L ΔSSTR = 0.50 ± 0.03, n = 10; wt +SSTR = 0.29 ± 0.01, n = 9; S218L +SSTR = 0.62 ± 0.04, n = 6; one-way ANOVA F_(3,28)_ = 28.34 p < 0.0001, post hoc Tukey test wt ΔSSTR vs. S218L ΔSSTR p = 0.0035 and wt +SSTR vs. S218L +SSTR p < 0.0001).

The plots of deactivation time constant against repolarization membrane potential (Fig. 3 F) show that the rate of change as a function of voltage was smaller in the S218L mutant channels (ΔSSTR: 24.28 ± 3.08 mV per e-fold change, n = 8; +SSTR: 19.75 ± 1.91, n = 6) than in the wt splice isoforms, reaching significance only for the ΔSSTR splice variant (wt ΔSSTR: 12.7 ± 1.26 mV, n = 7; wt +SSTR: 15.00 ± 1.10 per e-fold change, n = 9 one-way Welch’s ANOVA W_(3.0,13.12)_ = 5.54 p = 0.0112, post hoc Dunnett’s T3 test wt ΔSSTR vs S218L ΔSSTR p = 0.0359, wt +SSTR vs. S218L +SSTR p = 0.2774). Intuitively, the slow deactivation of mutant channels would allow a sustained Ca^2+^ entry after action potential repolarization, yet a shallower voltage dependence of channel closing might disrupt the effective contribution to the spike-triggered Ca^2+^ transients.

Together, our biophysical analyses indicate that the alternatively spliced SSTR tetrapeptide mainly affects voltage-dependence of Ca_v_2.1 gating and further distinctively modifies the effect of S218L mutation on Ca_v_2.1 activation, with nearly insignificant changes in voltage dependent inactivation.

### Molecular modeling of the S218L FHM-1 mutation in the + /Δ SSTR splice variants.

Conformational changes associated with voltage-driven gating occur concomitantly with changes in interactions between residues from adjacent segments. Mutations that strengthen or create new interactions are thought to stabilize particular conformational states resulting in altered voltage-dependent sensitivity [39]. Here, we find that the SSTR splice insertion or the S218L missense mutation results in a hyperpolarizing shift in the V_50_ of activation. A further strikingly shift in the voltage dependence of activation occurs when both changes are combined (e.g., +SSTR and S218L). Therefore, we wanted to explore whether the + / ΔSSTR alternative splicing event and the S218L missense mutation altered interactions between residues that might impact the transition between gating states. To investigate this, we examined the structural impact of the S218L mutation and SSTR variation using the recently published cryo-EM structure of Ca_v_2.1 [32].

**Fig. 4:**
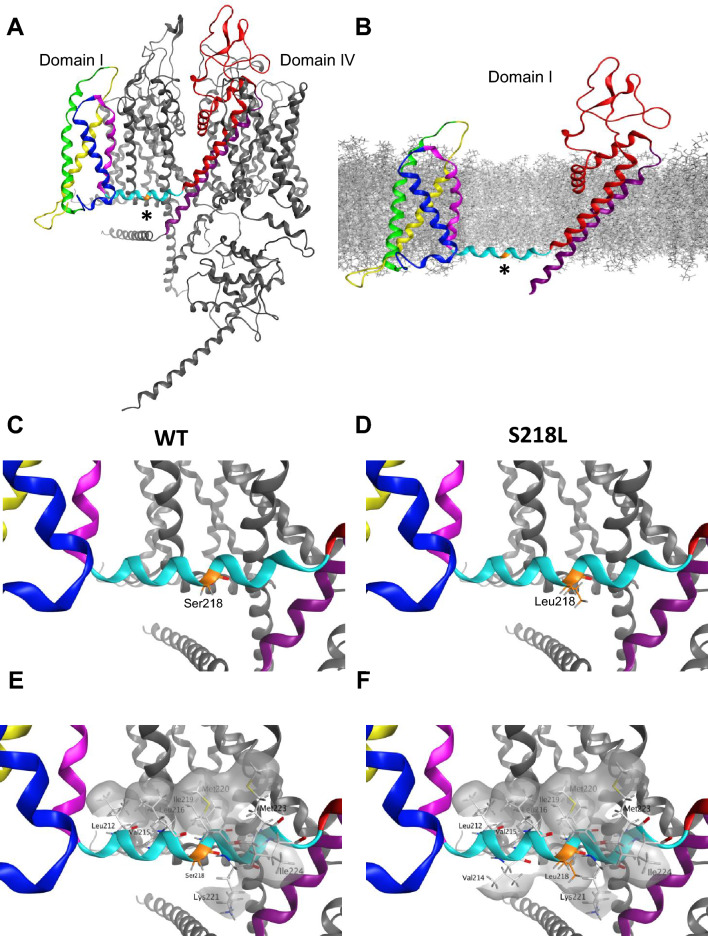
Molecular modeling of Cav2.1 S218L mutation depicts increased hydrophobicity in domain I S4-S5 linker. **A** Membrane view of Cav2.1 cryo-EM structure. VSD I is highlighted with S1 in yellow, S2 green, S3 blue and S4 pink, S4-S5 linker cyan, S5 red and S6 in purple. The location of Ser218 residue (*) is shown in orange. **B** Location of the Ser218 residue (*) with respect to modeled membrane in the isolated domain I. In a depolarized state, S4-S5 linker runs parallel to the inner surface the membrane. **C**–**D**) Orientation of WT Ser218 and Leu218 residues. Both Ser218 and Leu218 do not interact with any residues in neighboring domains. **E** The hydrophobic residues of the amphipathic domain I S4-S5 linker in WT are aligned on one side of the alpha helix, forming a hydrophobic patch (grey). **F** S218L mutation introduces a hydrophobic amino acid on the surface of the alpha helix facing the cytoplasm, increasing hydrophobicity with Val214 and Leu218

We first modeled the Ser218 to Leu218 substitution in the published Cav2.1 cryo-EM structural template that lacks the SSTR insertion [32]. The S218L mutation is located in the centre of the S4-S5 helical linker of domain I (Fig. 4A). In the Ca_v_2.1 structure, the domain I voltage sensor (VSD I) is in an ‘up’ state with a single S4 gating charge located below the occluding Phe residue. The S4-S5 linker is oriented parallel to the membrane (Fig. 4B), in accordance with the sliding-helix model where the position of the S4-S5 linker is determined by the movement of the S4 during gating. This is consistent with previous work using the structure of a voltage gated sodium channel in a resting and activated states showing that in the up position, the S4 pulls the S4-S5 linker towards the inner surface of the membrane [40]. We first analyzed whether the S218L mutation impacted interactions between residues in close proximity and in neighbouring segments. In the wildtype Cav2.1 structural template, the position of the domain I S4-S5 linker is stabilized by numerous interactions with neighboring residues in other segments from domain I and II that range from *van der Waals* interactions to hydrogen bonds. While approximately 65% of residues in the domain I S4-S5 linker are involved in various intersegment bonding interactions, the wildtype Ser218 residue does not appear to form any intersegment interactions in the conformational state captured by the cryo-EM structure (Fig. 4C). In our S218L model, Leu218 similarly did not form any intersegment interactions, nor did the mutation modify interactions between neighbouring residues (Fig. 4D), suggesting that the voltage dependent effects of S218L mutation do not stem from the strengthening of interactions between amino acids that stabilize the S4 ‘up’ conformational state.

As the S218L mutation involves the substitution of a polar amino acid to a hydrophobic Leu, we next generated surface maps of hydrophobic regions to investigate whether the mutation impacts hydrophobicity of the domain I S4-S5 linker region (Fig. 4E–F). The S4-S5 linker is amphipathic [41]; in domain I the hydrophobic residues Leu212, Val215, Leu216, Ile 219, Met220 and Met223 face the inner plasma membrane, while the residues Ser211, Val214, Lys217, Ser 218, Lys221 and Ile224 face the cytoplasm (Fig. 4E). Our model predicts that the S218L mutation leads to the formation of a larger hydrophobic region facing the cytoplasm formed by Val214, Leu218, Lys221 and Ile224 (Fig. 4F). During the transition from resting state to activated, as the domain I S4-S5 linker moves from the hydrophilic cytoplasm to the hydrophobic membrane [40], this enhanced hydrophobicity is predicted to make the transition more energetically favourable. Overall, our modeling indicates the S218L mutation likely stabilizes the domain I S4-S5 linker at the interphase between the inner membrane and the cytosol, potentially reducing the threshold for activation, and accounting for the major observed electrophysiology effect of a hyperpolarizing shift in the V_50_ of activation.

**Fig. 5:**
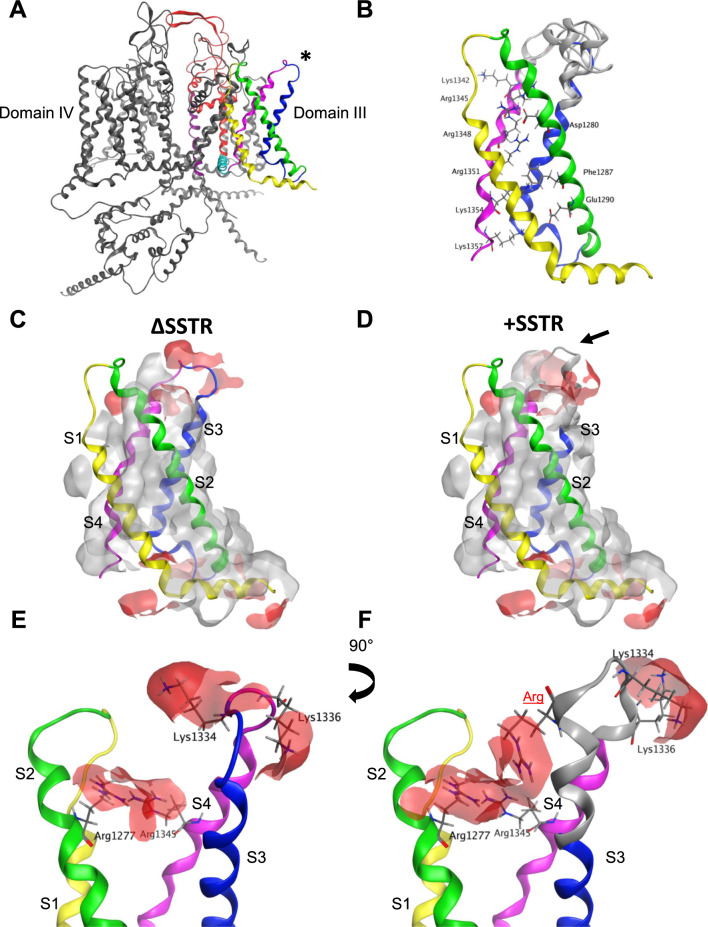
Inclusion of SSTR modifies positively charged surfaces surrounding VSD III. **A** Cav2.1 cryo-EM structure in ΔSSTR background shown in a membrane view. Domain III is highlighted with S1 in yellow, S2 green, S3 blue, S4 pink, S4-S5 cyan, S5 red and S6 purple. Location of SSTR insertion is highlighted by an asterisk. **B** Isolated VSD III with top 5 models of + SSTR splice variant shown in grey. Gating residues are highlighted. **C**–**D**) Hydrophobic (grey) and positively charged (red) patches in ∆SSTR (**C**) and + SSTR backgrounds (**D**). **E**–**F**) The inclusion of the tetrapeptide SSTR inserts a charged residue (Arg highlighted in red) that creates a positively charged region with Arg1277 in S2 and Arg1345 in S4 (**F**). The position of Lys1334 and Lys1336 are also shifted by SSTR insertion, changing the distribution of the positively charged region on the extracellular side of the domain III S3-S4 linker

We next examined how the insertion of the splice variant SSTR tetrapeptide might impact the loop structure of the Ca_v_2.1 domain III S3-S4 linker. The SSTR insertion occurs between T1330 and G1331 at the N-terminal region of the S3-S4 linker (Figs. 1A, 5A). Similar to VSD I, the S4 VSD III is in the up state (Fig. 5B). The structures predicted by the top 5 modeled loops show that the SSTR insertion either extends the alpha helix by approximately one and a half turns or increases the length of the domain III S3-S4 linker loop (Fig. 5B). The position of the S3 and S4 helixes were not substantially altered by the insertion, similar to previous modeling of a 19 residue S3-S4 linker splice variant insertion in the Ca_v_1.1 channel [39]. The analysis was performed in all five loops with the results shown corresponding to the representative loop displaying the highest percentage of common features. Given the close proximity to the domain III S4 voltage sensor, we first investigated whether the insertion of SSTR modifies interactions between gating charge residues. The modeling data predicts that the number or strength of interactions between gating charge residues is not significantly impacted by the SSTR insertion. Further, we did not find any significant changes in the strength of interactions in non-gating charge residues.

As the + e24a splice variant encodes for the insertion of three polar residues (2 Ser and 1 Thr) and a positively charged Arg, we also generated surface maps of hydrophobic and charged regions to investigate whether the +SSTR insertion indirectly modifies voltage sensing. Besides the gating charge residue Lys1342, in the wildtype ΔSSTR structure there are two positively charged regions that surround domain III S3 and S4: one region formed by Arg1277 and Arg1345 and the second by Lys1334 and Lys1336 (Fig. 5C, E). In our SSTR loop models, both positively charged regions were altered by the insertion. The inserted Arg extends the positively charged region formed by Arg1277 and Arg1345 (Fig. 5D, F). The modification of the domain III S3-S4 linker caused by the SSTR insertion also changed the orientation of several amino acids, including the positively charged extracellular residues Lys1334 and Lys1336, which are oriented away from each other in the ΔSSTR cryo-EM structure (Fig. 5E) and face the same direction in the +SSTR model (Fig. 5F). These newly charged regions are in proximity to key residues directly involved in gating. The inserted Arg appears to directly influence the positive surface formed by the gating charge residue Arg1345; while Arg1277 is separated by two residues from the negative counter charge Asp1280. Further, Lys1336 is separated by five residues from the first S4 gating charge Lys1342. These altered charged regions are likely to influence the local electric field sensed by these residues, suggesting the SSTR insertion impacts voltage sensitivity contributed by VSD III.

Lastly, we modeled the S218L mutation in the +SSTR background to understand how this combination might further shift the voltage dependence of activation. We did not find any additional change in the structure or interactions between residues in this model compared to each separate model. This may be due to the domain swapped arrangement of voltage gated Ca^2+^ channels [42], in which residues located in domain I and III have limited interactions with each other. As such, the combined functional effect of S218L and +SSTR may result from independent contributions of each respective VSD in shifting the voltage dependence of activation.

## Discussion

Post-transcriptional gene regulation of Ca^2+^ channels via alternative splicing generates multiple isoforms displaying distinctive cell- and tissue-specific expression patterns that can vary depending on the stage of development and in a species-dependent manner [43]. Alternative splicing of cassette exon e24a occurs in the orthologous mouse *Cacna1a* gene with transcripts carrying constitutive exons e24 and e25 showing higher levels of expression in the hippocampus, cerebellum and cortex than isoform e24a, which also showed negligible expression in the dorsal ganglion [31].

Splice-variant regional expression of Ca_v_2.1 transcripts has been previously shown to occur in human brain [5, 38]. Here, we employed RT-qPCR to examine the expression of + /e24a mRNAs in different regions of the human nervous system. Consistent with mouse tissue, our results showed a higher level of expression of e24a transcripts in the cerebellum with low levels of e24a expression in the spinal cord. In contrast, we observed higher e24a expression in the hippocampus and cerebral cortex. The regional differences between human and mouse could be due to methodological differences or to species-specific variation [43]. In humans, it is reasonable to assume that the differential splice-dependent effects of the S218L mutation have a significant functional impact on neuronal functioning across brain regions and development states. It is, however, important to note that the human tissue samples used in this study came from different individuals, including individuals with different ages, genders, and demographic groups, which may limit direct tissue-to-tissue comparisons. With caveats that the human data result from a small sample size and tissue samples from different individuals, we believe that the relative abundance of e24 variants may provide insight into why the FHM-1 pathological phenotypes linked to S218L manifest with different degrees of severity in a subset of nervous tissues.

Recent advances in cryo-EM have greatly increased our understanding of the spatial organization of molecular domains responsible for voltage-sensing, ion permeation and selectivity of Ca_v_ channels [44]. It has also made possible the use of homology-based structural models towards three-dimensional mapping of disease-related mutations that alter channel functioning [45]. The S218L is a well-characterized FHM-1 mutation with initial studies of recombinant hCa_v_2.1-S218L mutant channels showing a reduction in current density at more depolarized potentials as well as a hyperpolarizing shift in the activation curve [21]. These results were confirmed and expanded with evidence that the S218L mutation accelerates recovery from inactivation and increases accumulation of inactivation evoked by tonic depolarizations [20]. Further, the existence of differential splice-dependent effects of this pathogenic mutation on the biophysical properties of hCa_v_2.1 channels have also been reported [20]. Here, we employed the S218L mutation as a suitable model to examine whether combined changes in amino acid sequences, introduced by mutation and alternative splicing, affected spatial rearrangements or alter residue interactions, and that could provide insights into the splice-dependent functional consequences of the S218L mutation.

We chose to study hCa_v_2.1 splice variants produced by exclusion or inclusion of exon 24a, the latter of which electrophysiological properties have not been reported. Inclusion of exon 24a was previously described for the closely related Ca_v_2.2 channel from rat sympathetic ganglia [46] where the tetrapeptide SFMG apparently does not cause functional changes to channel properties [31]. In contrast, here we show for hCa_v_2.1 a pronounced shift in voltage dependence of activation associated with the SSTR insertion (Fig. 2D, E). Minor changes were also observed in the voltage dependence of channel activation and deactivation kinetics (Fig. 3).

Concerning the functional impact of the S218L mutation, the most notable changes were in the voltage sensitivity of activation when expressed in +SSTR containing channels, with a shift towards even more negative values compared to S218L ΔSSTR (Table 7) as well as a greater Ca^2+^ influx at potentials near the threshold of activation (Fig. 2B blue arrows). Consistent with previous studies and in agreement with the S218L mutation being associated with hyperexcitability [15], our data showed an increased subthreshold entry of Ca^2+^ in combination with a reduction of current density at depolarized potentials. It remains to be confirmed whether native neurons expressing the +SSTR variant with or without S218L exhibit a more severe phenotype than ΔSSTR expressing neurons. Similar changes in steady-state inactivation resulted from expression of the mutation in either isoform (Table 7), reducing the availability of channels within a similar range of membrane potentials (Fig. 2E). Whereas the splice insertion of +SSTR alone caused relatively subtle phenotypic changes in channel properties, the S218L +SSTR mutant channel displayed pronounced functional alterations compared to S218L ΔSSTR. It is intriguing that a small tetrapeptide insertion in a region facing the extracellular side of the membrane and located downstream of the S218L missense mutation causes such a strong impact on channel biophysical properties. We hypothesized that a mechanistic explanation might be revealed by three-dimensional modeling of the Ca_v_2.1 channel structure. Although the effects of the S218L mutation on gating might be explained by the location of the Serine residue in the domain I S4-S5 linker connecting the voltage-sensing domain with the pore module, we wondered whether the exacerbated effect of the mutation in the +SSTR isoform could be explained by a shift in the orientation of countercharge residues in the voltage sensor caused by the tetrapeptide insertion.

Missense mutations that alter the number and/or strength of intersegment interactions can impact the stability of conformational states, providing structural context for understanding effects on channel gating [27, 29, 47, 48]. In the current study, both the S218L mutation and SSTR insertion altered gating, revealed by a hyperpolarizing shift in the V_50_ of activation, suggesting that the energy barrier for pore opening is reduced thus requiring less depolarization for the outward displacement of the S4 helix. Intuitively, this is most likely to result from an increase in the stability of the activated state. Employing the Ca_v_2.1 cryo-EM structural template, we found relatively minor changes in the intersegment interactions. Rather, we found that charge and hydrophobicity of the modeled segments were significantly altered and predicted to impact stability regardless of conformational state. We first considered how the S218L mutation and insertion independently impact the structure of Ca_v_2.1 using the context provided by the S4 up state observed for VSD I and III [32, 33, 41, 49].

Interactions between residues in the S4-S5 linker and the S6 activation gate are thought to be important for coupling S4 movement and pore opening [41]. In the cryo-EM structure, Ser218 did not form any intersegment interactions, suggesting that this residue does not directly contribute to the stability of the domain I S4-S5 linker in the S4 up state. However, it is possible that Ser218 stabilizes another state. For instance, the hydrogen bonding capability of Ser218 may be important for the stability of the resting state, and that which would be lost with a Leu substitution. Future studies with a resting state structure of Ca_v_2.1 are required to explore this possibility. To understand how the hydrophobicity impacts the stability of the linker, we considered its movement during gating. The amphipathicity of the S4-S5 linker appears to be well conserved across voltage-gated ion channels [50]. This property is likely favourable for its role in electromechanical coupling, facilitating the movement of the S4-S5 linker during activation from the cytoplasm to the inner surface of the membrane [40]. In the S4 up state, the hydrophobic residues are aligned along the inner surface of the membrane whereas the polar residues face the cytoplasm. The substitution for the hydrophobic Leu218 disrupts this polar surface creating a hydrophobic region. We predict that this facilitates the movement of the S4-S5 linker away from the cytoplasm during activation, requiring less energy for the transition and reducing the threshold for activation as well as stabilizing its position near the inner membrane. This predicted stability of the S4 up state is in line with the increased open probability observed in previous work examining Ca_v_2.1 single channel properties [21], as well as the faster activation kinetics and slower deactivation kinetics observed in the current study. As our model only considers the state captured by the cryo-EM structure, whether the increase in hydrophobicity is sufficient to cause this gating shift requires further experimental validation. Despite this limitation, our modeling provides the first structural understanding for how the S218L FHM-1 mutation impacts P/Q-type channel voltage dependence and kinetics of Ca_v_2.1.

The structural basis for the shift in activation voltage dependence in the +SSTR splice variant is less clear. Since the insertion of four amino acids occurs at the N-terminal of the domain III S3-S4 linker, we examined whether interactions between residues in the S3 and S4 helixes were impacted. Positively charged gating residues in the S4 are stabilized by interactions with neighbouring residues including strong interactions with negatively charged residues. According to the sliding helix model, at rest the S4 is in the down state, pulled by the negative membrane potential. Upon depolarization, the S4 moves outward as gating charges exchange ion pair partners with negative countercharges [42, 51]. Here, finding a hyperpolarizing shift in the V_50_ of activation, we hypothesized that the tetrapeptide insertion would modify interactions between gating charges, stabilizing the S4 up state. However, our modeling data did not show any significant changes in interactions between gating and non-gating residues. One possibility is that the +SSTR insertion modifies interactions in gating states not captured by the structural template. For instance, a previous study involving a 19 residue Ca_v_1.1 splice variant in the domain IV S3-S4 linker found changes in interactions between residues in intermediate and activated states [39]. Our modeling data predicts that the smaller SSTR insertion leads to the redistribution of positively charged regions in domain III via the addition of the positively charged Arg residue and the reorientation of pre-existing residues. Given the proximity of these charged regions to key residues involved in gating, this may influence the local electric field sensed by these residues. Previous work in other voltage gated ion channels has shown that modifying the charge of the S3-S4 linker can influence the voltage dependence of activation [52, 53]. However, positively charged residues are thought to create repulsion with gating charges in the S4 helix, requiring increased depolarization for outward movement. Since the flexibility and length of the S3-S4 linker impacts activation of Ca_v_ channels, shaping the efficiency of S4 movement [54], we considered how the positively charged region would impact the movement of the domain III S3-S4 linker during gating. While the tetrapeptide insertion modestly increased the length of the linker, we propose that the more significant effect of the positively charged extracellular regions is to influence the movement of the domain III S3-S4 linker during gating. As the S4 helix moves outward during the transition to the activated state, the dynamic S3-S4 linker bends away from the outer vestibule [39]. The +SSTR insertion may promote the domain III S4 outward motion as the positively charged S3-S4 linker is moved away from the membrane and the repulsive S4 charges.

Finally, modeling the S218L in the +SSTR background did not further impact the channel structure, even though our electrophysiological findings show that the combination of S218L and +SSTR insertion causes an even larger shift in activation. Although all four VSDs have a similar structure, they do not contribute equally to voltage gating with key differences in the strength of the interactions and the displacement of the S4 helixes during transition between gating states suggested to underlie a functional heterogeneity of VSDs [55]. Our data supports the notion that both VSD I and VSD III each contribute to channel opening, and that each may independently facilitate transition to the activated state.

Ca_V_2.1 is highly expressed in the nervous system and differential temporal and spatial expression of splice variant isoforms is likely to result in subsets of P/Q-type proteins displaying heterogeneous phenotypes and distinct susceptibility to pathogenic mutations. Alterations in Ca_v_2.1-mediated Ca^2+^ signaling modulating neuronal excitability and synaptic strength has been suggested to underly an increased susceptibility to the induction and propagation of cortical spreading depression observed in hemiplegic migraine, recurrent seizures in epileptic patients, and cerebellar impairment associated to progressive ataxia in hereditary disorders involving CACNA1A mutations [56, 57] Thus, a differential distribution of transcript isoforms displaying distinct, splice dependent, functional effects of pathogenic mutations might contribute to the complex neurological phenotypes and clinical spectrum of comorbidities linked to CACNA1A mutations.

## Supplementary Information

Additional file 1.

Additional file 2.

Additional file 3.

## Data Availability

The raw data from the experiments in the current study are found in the supplementary information.

## Abbreviations

FHM-1: Familial hemiplegic migraine type 1
VSD: Voltage sensing domain
