# Supplementary material for: A structural analysis of the splice-specific functional impact of the pathogenic familial hemiplegic migraine type 1 S218L mutation on Cav2.1 P/Q-type channel gating
Source: Mol Brain. 2024 Nov 20;17:82. doi: 10.1186/s13041-024-01152-z (PMC11580629; doi:10.1186/s13041-024-01152-z)
Supplement: Supplementary file 1 — Additional file 1. [file 13041_2024_1152_MOESM1_ESM.pdf]

Supplementary figure 1. The overlap of membrane potential for steady state activation and inactivation (A: Δ SSTR; B: + SSTR) showed that there is a voltage interval at which a steady “window” current might occur (arrow). The window current, represented as the shaded areas below the curves in panels C (Δ SSTR) and D (+ SSTR), which correspond to the result of Boltzmann fitting, at a magnified scale. The values in Figure 2 panels D-E were used to replot the graphs shown here.
