# Supplementary figures and images for: A structural analysis of the splice-specific functional impact of the pathogenic familial hemiplegic migraine type 1 S218L mutation on Cav2.1 P/Q-type channel gating
Source: Mol Brain. 2024 Nov 20;17:82. doi: 10.1186/s13041-024-01152-z (PMC11580629; doi:10.1186/s13041-024-01152-z)

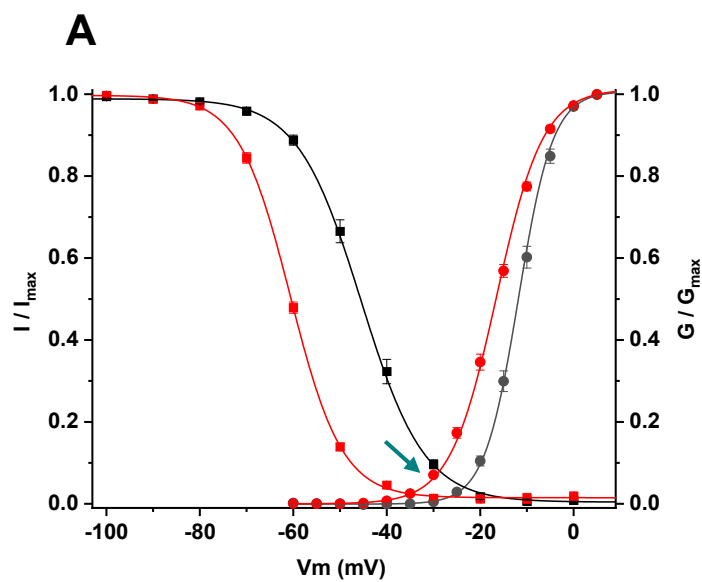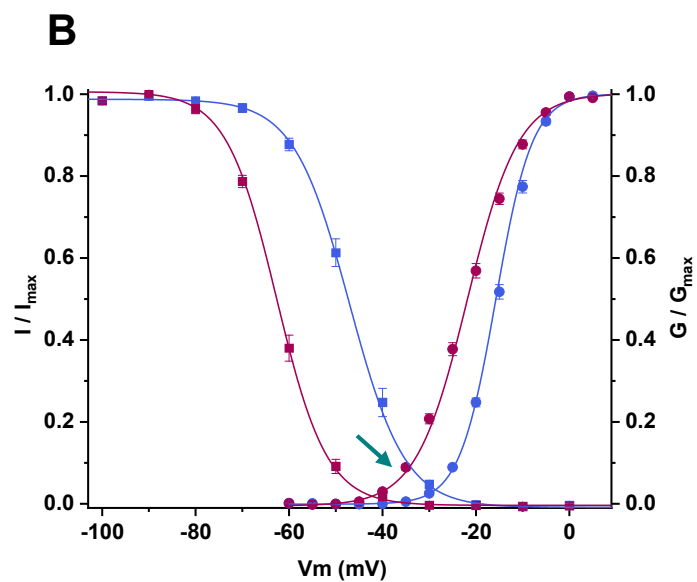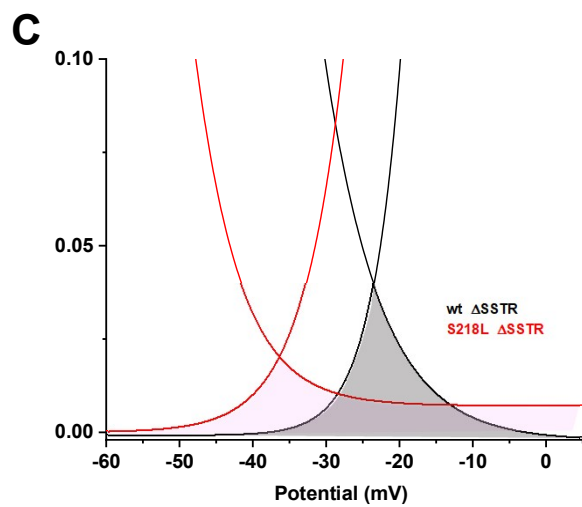

WT -SSTR  $V_{\max W} = -23.51$   
S218L -SSTR  $V_{\max W} = -36.37$

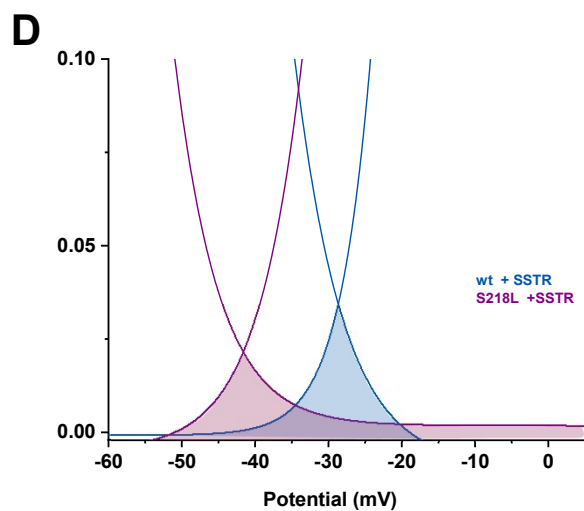

WT +SSTR  $V_{\max W} = -28.61$   
S218L +SSTR  $V_{\max W} = -41.71$

Supplement: Supplementary file 2 — Additional file 2. [file 13041_2024_1152_MOESM2_ESM.pdf]
